# Computational and Experimental Investigation of Micro-Hardness and Wear Resistance of Ni-Based Alloy and TiC Composite Coating Obtained by Laser Cladding

**DOI:** 10.3390/ma12050793

**Published:** 2019-03-07

**Authors:** Guofu Lian, Hao Zhang, Yang Zhang, Mingpu Yao, Xu Huang, Changrong Chen

**Affiliations:** 1 School of Mechanical & Automotive Engineering, Fujian University of Technology, Fuzhou 350118, China; gflian@mail.ustc.edu.cn (G.L.); zhanghao573@163.com (H.Z.); mpyao@smail.fjut.edu.cn (M.Y.); huangxu@fjut.edu.cn (X.H.); changrong.chen@fjut.edu.cn (C.C.); 2 School of Engineering + Technology, Western Carolina University, Cullowhee, NC 28723, USA; 3 Digital Fujian Industrial Manufacturing IoT Lab, Fuzhou 350118, China

**Keywords:** Ni35A + TiC composite, laser cladding, central composite design, micro-hardness, wear resistance

## Abstract

The influence of processing parameters on the micro-hardness and wear resistance of a Ni-based alloy and titanium carbide (TiC) composite cladding layer was studied. Mathematical models were developed to predict the micro-hardness and wear resistance of the cladding layer by controlling the laser cladding processing parameters. Key processing parameters were the laser power, scanning speed, gas flow, and TiC powder ratio. The models were validated by analysis of variance and parameter optimization. Results show that the micro-hardness is positively correlated with laser power and TiC powder ratio, where the TiC powder ratio shows the most significant impact. The wear volume decreased with an increasing TiC powder ratio. The targets for the processing parameter optimization were set to 62 HRC for micro-hardness and a minimal volume wear. The difference between the model prediction value and experimental validation result for micro-hardness and wear volume were 1.87% and 6.33%, respectively. These models provide guidance to optimize the processing parameters to achieve a desired micro-hardness and maximize wear resistance in a composite cladding layer.

## 1. Introduction

Laser cladding is a surface modification technology that is used to enhance the mechanical properties of a substrate. In this process, a thin layer of metal powder is placed on the surface of the substrate and both the powder and substrate are melted with a laser beam to form a cladding layer [1,2]. Due to its modest heat affected zone, small-scale deformation, low dilution rate, and rapid melting and solidification, laser cladding has been widely applied to repair and remanufacture crank shaft and other high value parts in the aerospace, marine, petroleum, and chemical industries [3,4,5]. 

Recent development in laser cladding powders and surface coatings has been extended beyond traditional materials to include composite materials. For instance, NiCrSiB series alloys are extensively utilized in industry due to their exceptional corrosion, wear and fatigue resistance, and cost efficiency [6,7,8]. Within this series, most of the research has focused on Ni60A powder. However, Ni60A has high brittleness, which could lead to a high possibility of fracture. Ni35A possesses high plasticity but low hardness. However, its hardness could be improved by incorporating add-on materials [9]. TiC has a high melting point, high hardness, outstanding wear resistance, and a low coefficient of friction. Thus, TiC has been frequently adopted to enhance material properties [10,11]. Because of the phenomenal properties, research has been concentrated on combining nickel-based alloys and TiC to form composite cladding materials.

Bakkar et al. investigated the microstructure, micro-hardness, wear resistance, and corrosion resistance of different volume percentages of TiC/Inconel 625 nickel-based composite alloys. They found that the micro-hardness and wear resistance of these type of composites could be improved by adding an appropriate volume fraction of TiC [12]. Muvvala et al. conducted laser cladding experiments with different weight percentages of TiC/Inconel 718 nickel-based composite alloy powder and investigated the impact of processing parameters on the molten pool and structural properties. Analyzing the result illustrated that optimizing processing parameters could effectively disperse fine TiC particles, which enhanced hardness and wear resistance of the cladding layer [13]. Liu et al. produced a TiC enhanced nickel-based composite coating. They concluded that the phase and distribution of TiC were affected by convection in the molten pool [14]. Saroj et al. created a TiC-Inconel 825 composite coating employing a Tungsten Inert Gas (TIG) cladding method and discovered the impact of different TiC percentages (20%, 40%, and 60%) and processing currents on the coating morphology, micro-hardness, and wear resistance. Because of the superior wettability and binding between nickel-based alloys and TiC, the coating hardness reached 1100 HV_0.05_, and the wear resistance improved seven times over that of the substrate [15]. Sahoo et al. also utilized TIG cladding to build TiC-Ni composite coating on an AISI304 steel substrate. The results exhibited outstanding binding between the coating and substrate, the hardness reached 1300 HV_0.05_, and the wear resistance increased 70 times with TiC-Ni composite coating when compared to the substrate [16].

Existing research on a nickel-based alloy and TiC composite material are primarily focused on the influence of different TiC ratios and processing parameters on the structural properties, micro-hardness, and wear resistance of the coating since micro-hardness and wear resistance are two important factors evaluated in industrial applications. However, predicting and controlling the cladding layer micro-hardness and wear resistance by manipulating the laser cladding processing parameters and TiC ratio have rarely been explored. In this paper, mathematical models are developed to predict the micro-hardness and wear resistance of the cladding layer by controlling the laser cladding processing parameters and TiC powder ratio. 

## 2. Materials and Methods

**Figure 1:**
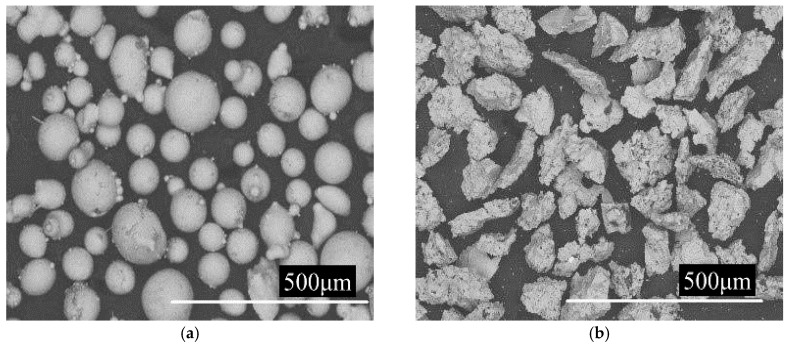
(**a**) Morphology of Ni35A powder. (**b**) Morphology of TiC powder.

AISI/SAE 1045 steel was selected as the substrate with a size of 40 mm × 20 mm × 5 mm. The laser beam diameter was adjusted to 4 mm. Cladding powder was made from Ni35A and TiC powder with a particle size ranging from 48 μm to 106 μm. The elemental composition and morphology of Ni35A and TiC power are shown in Table 1 and Figure 1.

**Figure 2:**
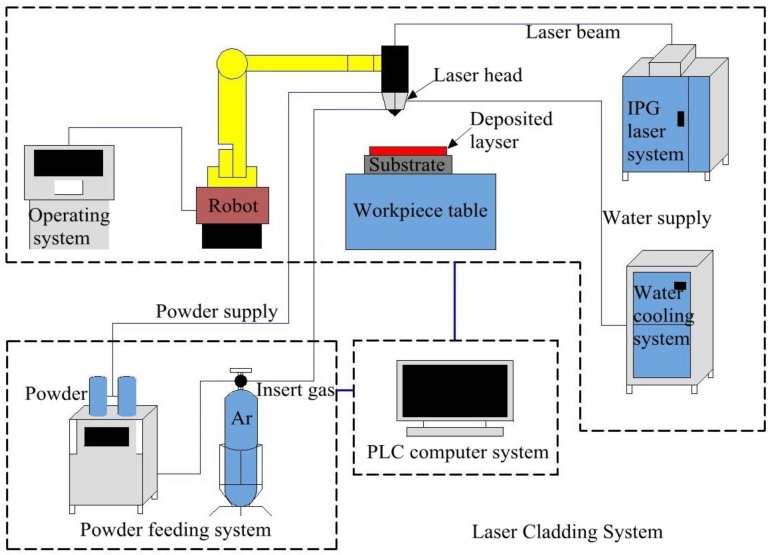
Laser cladding system.

Figure 2 explains the laser cladding system, which includes a laser system (YLS-3000, IPG, Burbach, Germany), laser cladding nozzle with 300 mm focal length (FDH0273, Lasermech, Novi, MI, USA), industrial robot (M-710iC/50, FANUC, Yamanashi, Japan), water cooling system (TFLW-4000WDR-01-3385, Sanhe Tongfei, Sanhe, China), powder feeding system (CR-PGF-D-2, Songxing, Fuzhou, China), control system (PLC, Mitsubishi, Japan), and laser pulse control system (SX14-012PULSE, IPG, Burbach, Germany). Argon gas was used to protect the material during the cladding process.

Before cladding, the 1045 steel substrate surface was cleaned with ethanol. The Ni35A and TiC powder was mixed in a MITR--YXQM-2L ball mill machine (MITR, Changsha, China) for 30 min at a speed of 300 rpm and then placed in a vacuum dryer for an additional 30 min at a temperature of 120 °C. After completion of the laser cladding, the sample was processed by cutting, setting, grinding, and polishing. Then the sample was immersed in 4% nitric acid and alcohol mixture for 30 s. An MVA-402TS micro-hardness tester (HDNS, Shanghai, China) was utilized to measure the micro-hardness with a 500 g-force applied for a 30-s duration. The microstructure was observed using a scanning electron microscope (SEM) TM3030Plus (HITACHI 550I, Tokyo, Japan). In addition, element analysis was performed using an energy-dispersive X-ray spectroscopy (EDS) system (A550I, IXRF, Austin, TX, USA). The wear resistance was examined with a UMT-2 high load scratch tester (Bruker, Billerica, MA, USA). The X-ray diffraction (XRD) analysis was conducted with Ultima IV XRD systems (Rigaku Corporation, Tokyo, Japan). 3D morphology of the abrasion surface was obtained using white light interferometry. Afterwards, the width and depth of the worn area was measured. The average wear off area was obtained by repeating the previously mentioned measurement in five different locations. In the end, wear volume was calculated by multiplying the average wear off area by the scratching distance. The wear volume was used to evaluate wear resistance in which lower wear volume indicates greater wear resistance. The friction coefficient was measured every 0.1 s over a duration of 60 min. Conditions for the wear test are shown below in Table 2.

Respond surface methodology (RSM) is an optimization method that can be used to build a mathematic model between output and input variables. In this study, the central composite design (CCD) module in RSM was selected. The experimental design matrix contained four factors and five levels. The four factors were laser power (LP), scanning speed (SS), gas flow (GF), and TiC powder ratio (PR). The variables for the factors were set to 0, ±1, and ±2 in the Design Expert software (Version 10.0). Then the interaction respond value between input and output was obtained with the CCD design. The intent of RSM is to build a statistical prediction model based on experimental results. Afterwards, analysis of variance was utilized to analyze the model [17]. Multiple regression analysis was employed to build and analyze the model between the input parameters and the output. The polynomial regression function is shown in Equation (1) below [18], where y is the response value and *β*_0_ is the intercept factor. The coefficients for the linear term, interaction term, and quadratic term are *β_j_*, *β_ij_*, *β_jj_*, respectively. Additionally, *x_i_* and *x_j_* represent the processing parameter, *k* is the number of factors, and ε is the residual.

(1) $$y=\beta_{0}+\sum_{j=1}^{k}\beta_{j}x_{j}+\sum_{i,j=1}^{k}\beta_{ij}x_{i}x_{j}+\sum_{j=1}^{k}\beta_{jj}x_{j}^{2}+\varepsilon$$

The laser cladding processing parameter variables are exhibited in Table 3. Experimental design and results are shown in Table 4.

## 3. Results and Discussion

### 3.1. Analysis of Variance

Variance analysis of micro-hardness and wear volume (Table 5 and Table 6) was used to examine the reasonableness of the selected model. Note that, for the micro-hardness model, its P-value is less than 0.0001 and the lack of fit is larger than 0.05. These results indicate that there is only a 0.0001 probability of causing interference. The adequate precision (signal-to-noise ratio) value of 29.479 is larger than 4, which indicates that model accuracy is satisfied. The closer the value of R-Square to 1 is, the better fit the model is. The R-Square value of this model is 0.9447. In addition, the Adjusted R-Square and Predicted R-Square values are both close to 1 and the difference between these two values is 0.0176, which is less than the needed value of 0.2 [18]. These results demonstrate that this model has a high level of fit and could be used to precisely predict the correlation between processing parameters and micro-hardness. Similarly, the wear volume model also meets these expectations. 

In Table 5, it can be seen that the TiC powder ratio and its quadratic term are the dominant factors in the micro-hardness model. Laser power has a moderate effect and the effect of scanning speed is negligible. It is evident from Table 6 that the TiC powder ratio is also the most significant parameter in the wear volume model. The micro-hardness and wear volume models are shown below in Equations (2) and (3), where LP is laser power, SS is scanning speed, and PR is the TiC powder ratio. LP × SS, LP × SS, and SS × PR are the interaction terms. The quadratic term of the powder ration is represented by PR^2^.

(2) $$\begin{array}{cc} \mathrm{Microhardness}= & 58.20639-12.09167\times \mathrm{LP}-1.60125\times \mathrm{SS}+0.42509\times \mathrm{PR}+1.61250\\ & \times \mathrm{LP}\times \mathrm{SS}+0.31438\times \mathrm{LP}\times \mathrm{PR}-0.22687\times \mathrm{SS}\times \mathrm{PR}-4.2218\times 10^{-3}\\ & \times {\mathrm{PR}}^{2} \end{array}$$

(3) $$\begin{array}{cc} \mathrm{Wear}\;\mathrm{Volume}= & 1.33239\times 10^{5}-85232.58542\times \mathrm{LP}-16189.90302\times \mathrm{SS}-736.06472\\ & \times \mathrm{PR}+11585.69688\times \mathrm{LP}\times \mathrm{SS}+207.21641\times \mathrm{LP}\times \mathrm{PR}+36.73658\times \mathrm{SS}\\ & \times \mathrm{PR}+0.58615\times {\mathrm{PR}}^{2} \end{array}$$

### 3.2. Analysis of the Micro-Hardness Model

**Figure 3:**
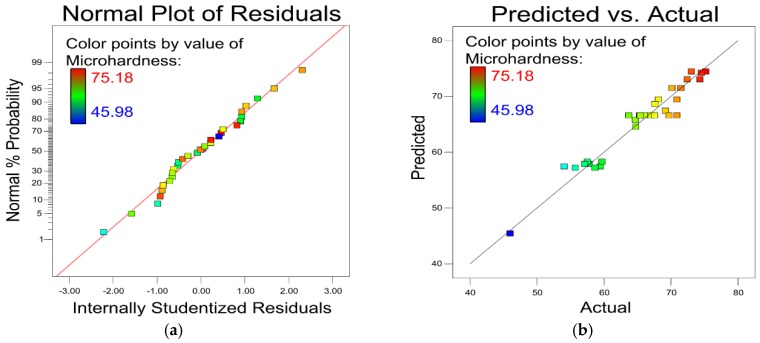
(**a**) Residual analysis of micro-hardness. (**b**) Comparison of predicted and actual micro-hardness.

The plot of the residual factor for the micro-hardness model is shown in Figure 3a. The almost linear distribution of the plot indicates an exceptional fit to the model. Figure 3b shows the small-scale error between the predicted and actual micro-hardness experimental values, which demonstrates this model has high prediction accuracy.

**Figure 4:**
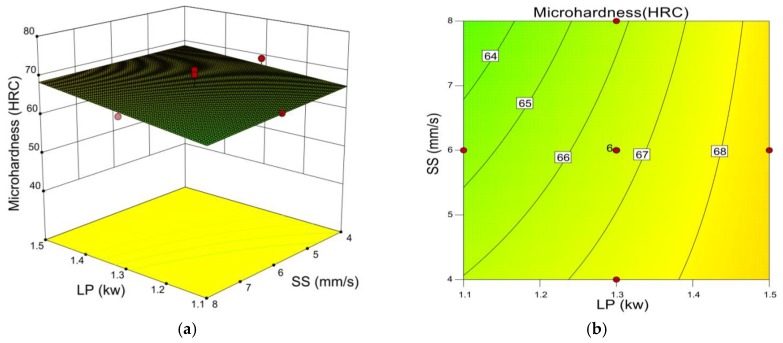
(**a**) 3D influence of laser power and scanning speed on micro-hardness. (**b**) Contour line of laser power and scanning speed on micro-hardness.

**Figure 5:**
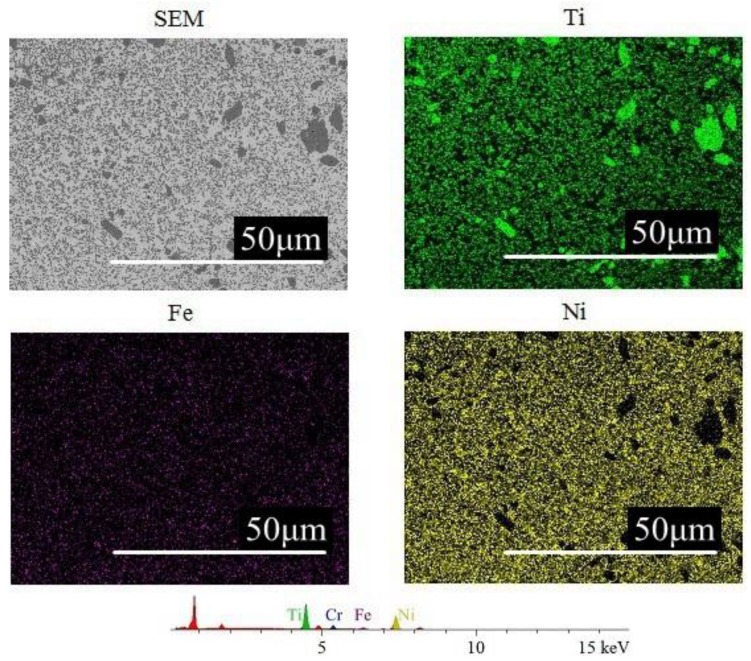
Element analysis on the cladding layer cross section (1.1 kW laser power).

**Figure 6:**
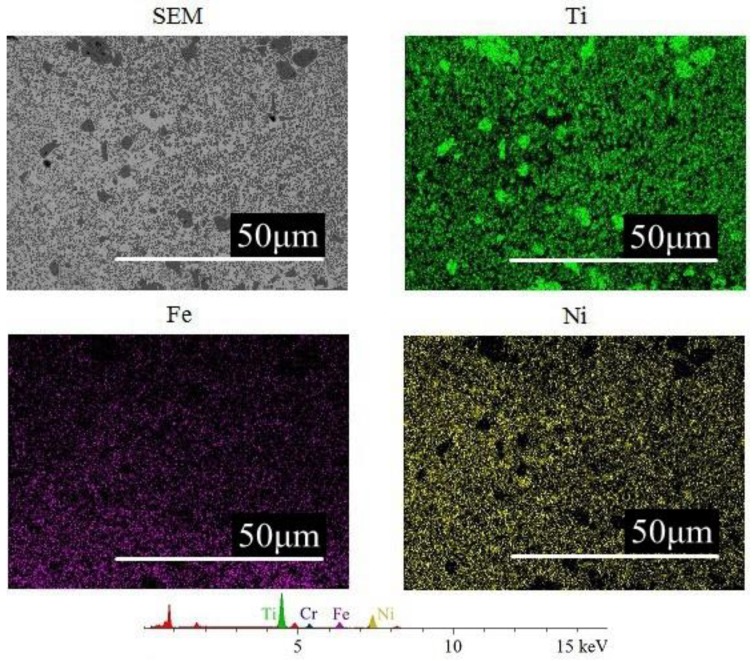
Elements analysis on the cladding layer cross section (1.5 kW laser power).

Figure 4 shows laser power to be positively correlated to micro-hardness and scanning speed to be negatively correlated with micro-hardness. This phenomenon is due to the fact that laser power defines the amount of energy absorbed by the powder during laser cladding. With other conditions being the same, higher laser power corresponds to more energy being absorbed. Element analysis by EDS of the cladding layer cross section was performed on samples made with 6 mm/s scanning speed, 1200 L/h gas flow, 40% TiC powder ratio, and laser power of 1.1 kW (Figure 5) and 1.5 kW (Figure 6). Iron content in the cladding layer increased with the higher laser power. The distribution of iron is relatively even in Figure 5 due to the effects of elemental diffusion. However, the distribution of iron in Figure 6 decreases from bottom to top, which results from more iron being diffused into the cladding layer since higher laser power promotes more intensive elements diffusion. A higher laser power causes an increase in TiC melting and an increased possibility of nucleation, which creates smaller crystallite and increases the micro-hardness [19,20,21]. In addition, the scanning speed controls the amount of time the cladding powder is exposed to the laser beam. With the same laser power, a lower scanning speed increased the amount of energy delivered to the cladding material. This condition helps the melting of TiC and improves the micro-hardness of the cladding layer. On the contrary, a higher scanning speed reduces the exposure time, which reduces the energy delivered to the cladding powder and has an adverse effect on melting of TiC. Thus, increased scanning speed decreases micro-hardness.

**Figure 7:**
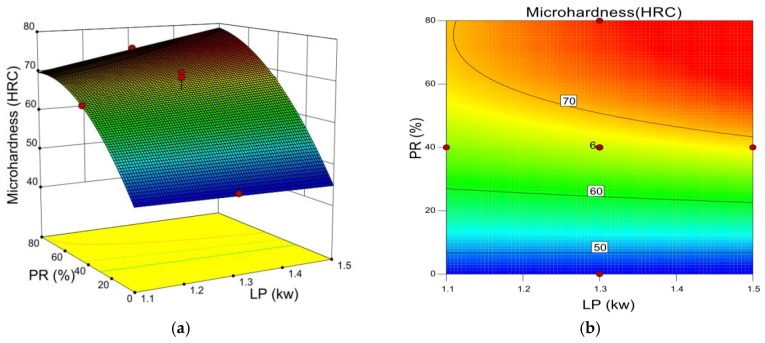
(**a**) 3D influence of laser power and TiC powder ratio on micro-hardness. (**b**) Contour line of laser power and TiC powder ratio on micro-hardness.

Figure 7 shows how the interaction of laser power and the TiC powder ratio influences micro-hardness. It is evident that the TiC powder ratio has stronger influence on the cladding layer micro-hardness than the laser power, which is consistent with Table 5. The micro-hardness increases with the TiC ratio because a known property of TiC is that it can serve as a hardness enhancement compound.

**Figure 8:**
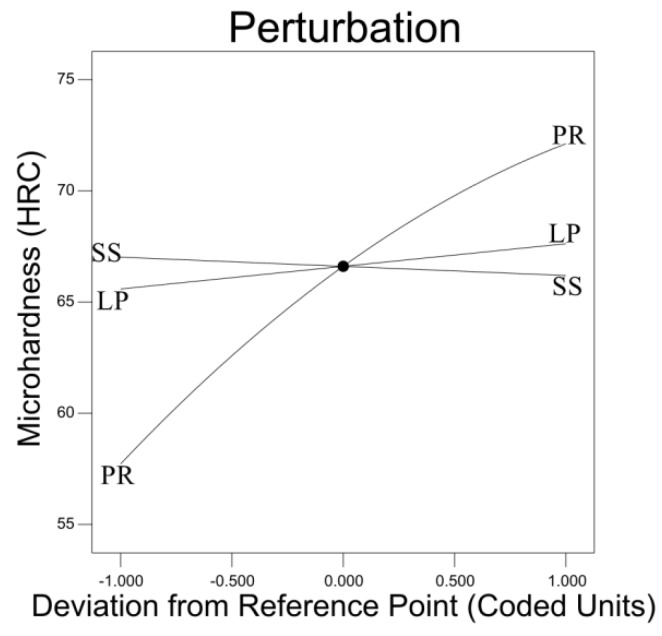
Impact of different parameters on micro-hardness.

Figure 8 displays the impact of different parameters on the micro-hardness. The most significant impact on the micro-hardness is the TiC powder ratio. Laser power is linearly related to micro-hardness and positively correlated. Scanning speed is also linearly related to micro-hardness, but has a negative correlation.

### 3.3. Analysis of the Wear Volume Model

**Figure 9:**
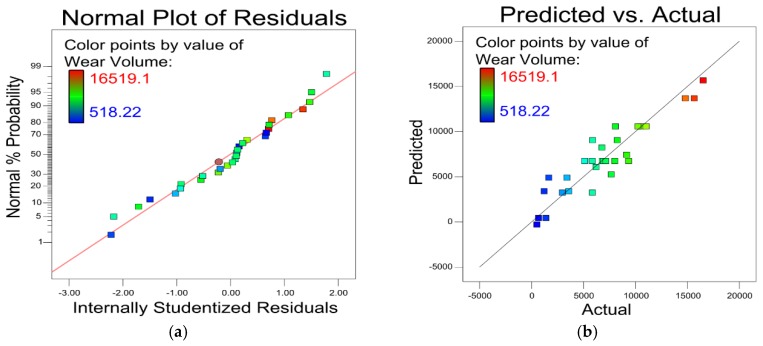
(**a**) Residual analysis on wear volume. (**b**) Comparison of predicted and actual wear volume.

From Figure 9, it can conclude that the wear volume model demonstrates exceptional prediction accuracy. The model could effectively account for different processing parameters and accurately predict the wear volume as seen by the nearly linear distribution in Figure 9a and error scale in Figure 9b.

**Figure 10:**
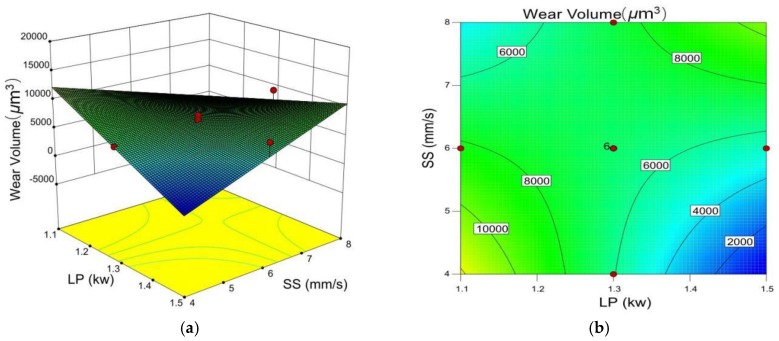
(**a**) 3D influence of laser power and scanning speed on wear volume. (**b**) Contour line of laser power and scanning speed on wear volume.

The wear volume of the cladding layer increases as the laser power decreases and the scanning speed increases (Figure 10). Lower laser power results in less energy being absorbed by the cladding layer. In addition, the grain size was measured with the Nano Measurer, where the average grain size is 14.62 μm and 18.66 μm in the cladding layer obtained with 6 mm/s scanning speed, 1200 L/h gas flow, 40% TiC powder ratio, and laser power 1.5 kW and 1.1 kW, respectively. Therefore, a fine crystal grain could be obtained with higher laser power while other parameters remain the same. In addition, according to the Hall-Petch relation, fine crystal grains will also contribute to increased cladding layer micro-hardness [22]. Increasing laser power could aid in grain refinement and nucleation, which decomposes more TiC and promotes the diffusion of fine grains. Therefore, better wear resistance could be achieved with larger laser power.

**Figure 11:**
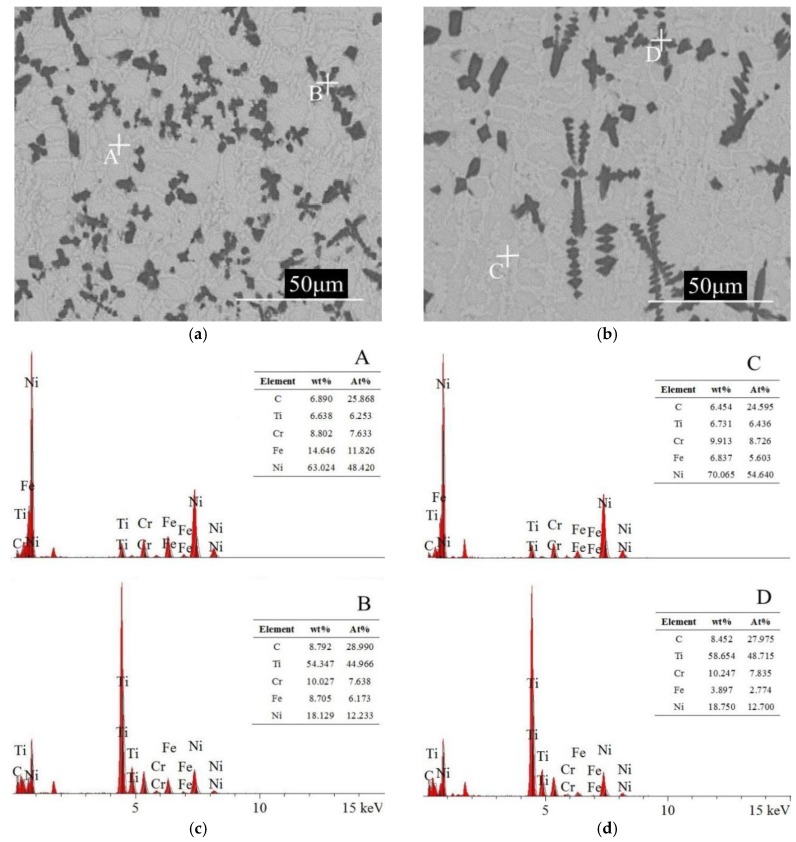
Structure of the cladding layer made from (**a**) 5 mm/s scanning speed. (**b**) 7 mm/s scanning speed. Element analysis of the cladding layer made from (**c**) 5 mm/s scanning speed. (**d**) 7 mm/s scanning speed.

The cladding layers shown in Figure 11 were obtained using a 1.2 kW laser power, 1400 L/h gas flow, 60% TiC powder ratio, and scanning speeds of 5 mm/s and 7 mm/s. The lower scanning speed results in a longer exposure time, which creates a more uniformed structure and leads to the improved micro-hardness and wear resistance. EDS was used to conduct the element analysis in two areas for each sample. The nickel percentage was much higher in the nickel-based structures area A and C. Titanium percentage was higher in the TiC dendrite structures area B and D. The iron percentage in the cladding layer at the 7 mm/s scanning speed was lower than at the 5 mm/s scanning speed. A higher percentage of iron will enhance solid solution strengthening, which improves the micro-hardness and wear resistance of the cladding layer [22]. Therefore, a faster scanning speed will lead to a lower wear resistance and a larger wear volume.

**Figure 12:**
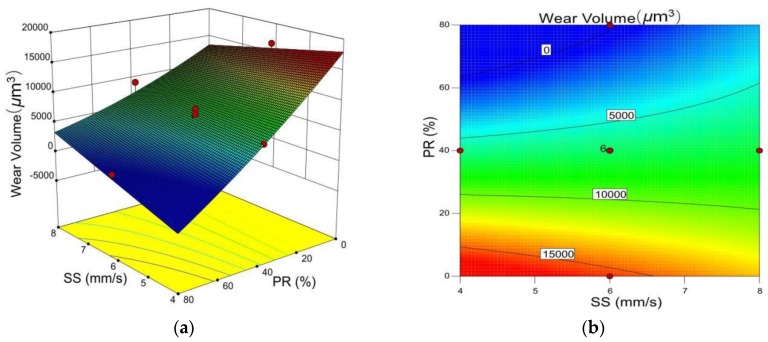
(**a**) 3D influence of scanning speed and the TiC powder ratio on wear volume. (**b**) Contour line of scanning speed and TiC powder ratio on wear volume.

It can be observed from Figure 12 that the wear volume increases as the TiC powder ratio decreases. Ni35A powder contains elements of Fe, Cr, B, and Si. Rapid melting and solidification during the laser cladding process causes elemental diffusion resulting in super-saturation leading to solid solution strengthening. With the increase of the TiC powder ratio, second phase and solid solution strengthening significantly increases due to the mixture of the ionic bond, the covalent bond, and the metallic bond within the grain structure. This causes a significant improvement of wear resistance [23,24,25].

**Figure 13:**
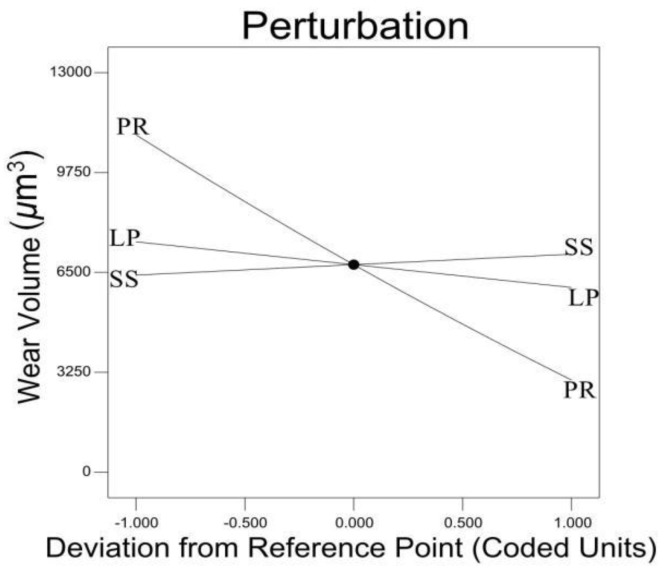
Impact of different parameters on wear volume.

Figure 13 demonstrates the scanning speed positively affected the wear volume and the laser power and TiC powder ratio negatively affects the wear volume. The TiC powder ratio has the most significant impact. All of these three processing parameters display a linear relation with wear volume.

### 3.4. Phase Analysis

**Figure 14:**
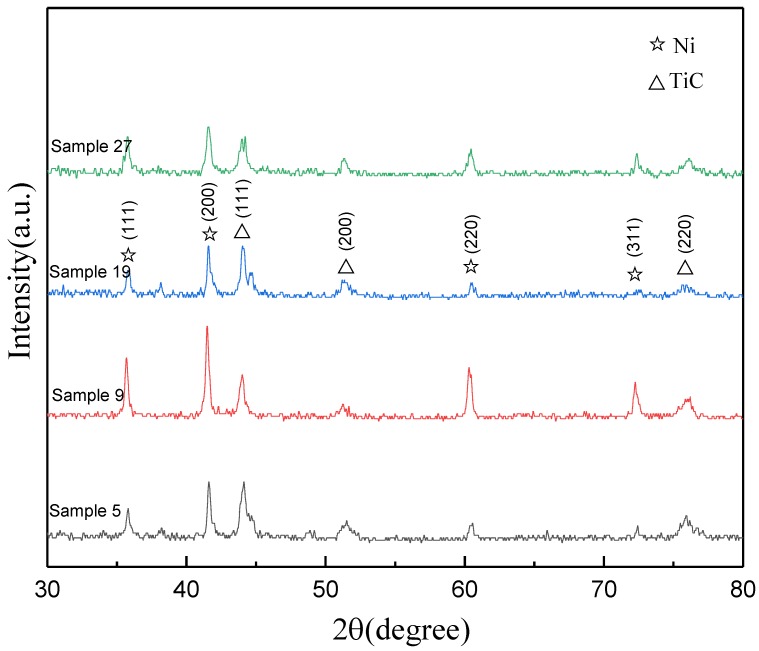
XRD patterns of different samples.

Figure 14 shows the XRD diffraction patterns of four randomly selected samples since it is assumed that no phase change would be evolved during laser cladding and only peak intensity would vary. It indicates that the principal phases in the cladding layers are TiC and Ni. The TiC was identified as the cubic structure Khamrabaevite (JCPDS: 65-0242). The nickel was identified as the cubic structure Ni (JCPDS: 65-0380). From the same results observed in all the samples, it indicates that phase compositions remain unchanged as the raw material. Moreover, the laser cladding process is mainly a physical reaction and the chemical reaction is negligible.

### 3.5. Processing Parameter Optimization and Model Validation

The criteria and limits of the processing parameters and the response of the optimization are listed in Table 7. Since the micro-hardness and wear volume are important factors to evaluate the quality of the cladding layer, their importance levels are both assigned as five (within range 1–5, larger number, higher importance). The hardness requirement for regular tools of 62 HRC [26] was adopted as the target for micro-hardness. The goal for wear volume was to achieve a minimum value in order to attain higher wear resistance.

The optimal parameters were found to be a laser power of 1.5 kW, scanning speed of 4 mm/s, gas flow of 1408.558 L/h, and TiC powder ratio of 26.964% (Table 8). Based on limitation of the equipment set point accuracy, the test parameters of the validation experiment were set as a laser power of 1.5 kW, scanning speed of 4 mm/s, gas flow of 1400 L/h, and TiC powder ratio of 27%. The micro-hardness and wear volume of the predicted and experimentally validated values are also shown in Table 8. The error of the prediction was calculated to be 1.87% for micro-hardness and 6.33% for wear volume.

**Figure 15:**
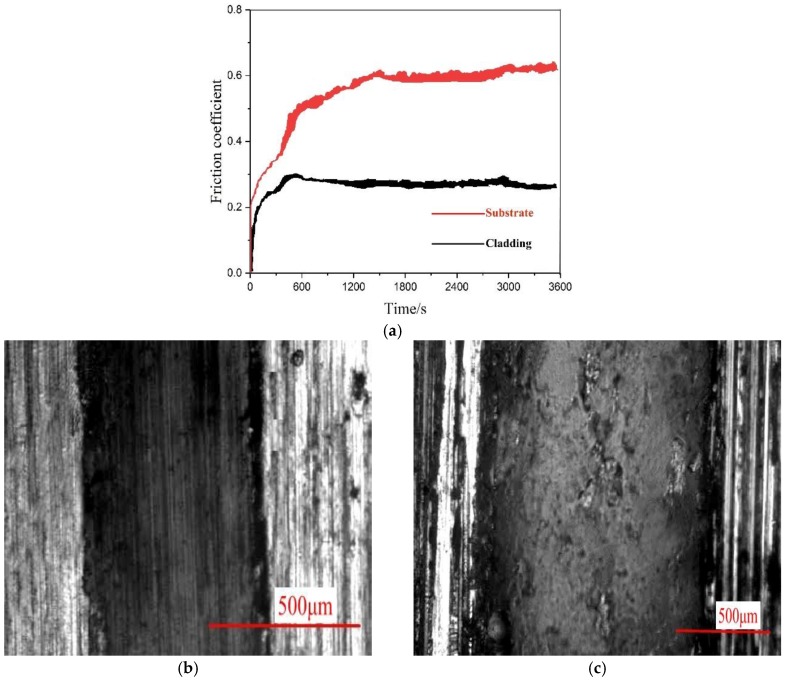
(**a**) Friction coefficient curve of the cladding layer and substrate. (**b**) Wear morphology of the cladding layer made with optimized parameters. (**c**) Wear morphology of the substrate without a cladding layer.

Figure 15a shows that the friction coefficient of the cladding layer to increase for the first 10 min then stabilizes at a value of approximately 0.25. The friction coefficient of the substrate also increases over time and stabilizes around 0.61. The difference in the coefficient of friction between the cladding layer and the substrate can be explained by the TiC powder ratio of 27% in the cladding layer, which promotes more second-phase strengthening and improves its wear resistance. The furrow wear and a large amount of peeling observed in the substrate (Figure 15b) was not observed in the cladding layer (Figure 15c) due to the solid solution strengthening from TiC and second-phase strengthening. Second-phase strengthening had protected the pressing of the grinding head with only slight scratches being observed. It demonstrates that application of a cladding layer with an optimized parameter can be an effective surface modification method.

**Figure 16:**
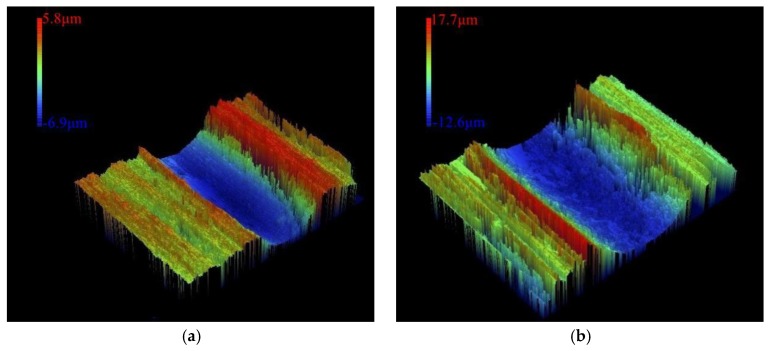
(**a**) Wear on the cladding layer. (**b**) Wear on the substrate.

The 3D morphology of the worn area is shown in Figure 16. The wear volume of AISI/SAE 1045 steel was 20,114.36 μm^3^ (Figure 16a) and the wear volume of the cladding layer with optimized parameters was 3261.57 μm^3^ (Figure 16b). Under the same testing conditions, the wear volume of the substrate enhanced by the cladding layer was reduced 83.8% compared with the substrate. The cladding layer improved the wear resistance 6.17 times when compared to the AISI/SAE 1045 steel substrate without cladding.

## 4. Conclusions

This research developed the models relating the laser cladding processing parameters (laser power, scanning speed, gas flow, and TiC powder ratio) of a composite material cladding layer to the micro-hardness and wear volume through response surface methodology. A validation experiment conducted with optimized processing parameters verified the reliability of these models. These models provide guidance for processing parameters optimization, composite material cladding layer property prediction, and control. Conclusions can be drawn as follows:

1. A near linear relationship exists between micro-hardness and processing parameters. Micro-hardness is primarily affected by the TiC powder ratio. A higher micro-hardness in the cladding layer can be obtained by increasing the TiC powder ratio, increasing the laser power, and decreasing scanning speed.
2. The correlation between wear volume and processing parameters also appears to be linear. TiC powder ratio has a major influence on the wear volume of the cladding layer. Increasing laser power and TiC powder ratio and decreasing scanning speed could achieve smaller wear volume.
3. Enhancing the surface of the substrate by applying a cladding layer with optimal processing parameters increased the wear resistance by approximately 6.17 times and reduced the wear volume by about 83.8%.

## Figures and Tables

**Table 1:** Elemental composition (wt.%) of Ni35A and TiC powder.

Powder	Element (wt.%)									
	C	Si	O	Fe	Cr	B	T.C	F.C	N	Ni
Ni35A	0.32	3.35	<0.05	2.75	7.75	1.65	-	-	-	Rest
TiC	-	0.02	0.5	0.08	-	-	>18.8	<0.5	0.5	-

**Table 2:** Scratch testing parameters.

Parameters	Unit	Specifications
Friction pair	mm	Cemented Carbide—Φ 6 mm
Force	N	35
Speed	mm/s	10
Distance	mm	4
Duration	min	60
Mode	-	Reciprocating
Temperature	°C	Room temperature

**Table 3:** Laser cladding processing parameter variables.

Variables	Notation	Unit	Levels of Input Variables					
			Code	−2	−1	0	1	2
Laser power	LP	kW	Actual	1.1	1.2	1.3	1.4	1.5
Scanning speed	SS	mm/s		4	5	6	7	8
Gas flow	GF	L/h		800	1000	1200	1400	1600
TiC powder ratio	PR	wt.%		0	20	40	60	80

**Table 4:** CCD experimental design and results.

Run	LP (kW)	SS (mm/s)	GF (L/h)	PR (wt.%)	Micro-Hardness (HRC)	Wear Volume (μm^3^)
1	1.4	5	1400	60	75.18	1386.04
2	1.2	7	1000	20	55.70	8076.80
3	1.3	6	800	40	67.02	5840.25
4	1.4	7	1000	20	57.50	10,499.10
5	1.4	5	1000	20	57.78	5877.28
6	1.3	6	1200	40	63.68	5116.18
7	1.4	7	1000	60	74.32	1659.55
8	1.4	5	1000	60	73.04	669.07
9	1.2	5	1000	60	70.14	3582.03
10	1.4	5	1400	20	57.12	8248.70
11	1.2	5	1400	60	71.48	1221.86
12	1.3	6	1200	80	74.54	518.22
13	1.2	5	1000	20	59.44	14,804.70
14	1.3	6	1200	40	66.06	6982.62
15	1.3	6	1200	40	70.88	6938.96
16	1.4	7	1400	60	72.42	3408.27
17	1.2	7	1000	60	70.88	5854.99
18	1.3	6	1200	40	65.30	6821.46
19	1.2	5	1400	20	54.04	15,654.50
20	1.2	7	1400	20	58.64	10,248.70
21	1.4	7	1400	20	59.70	11,030.70
22	1.3	6	1200	0	45.98	16,519.10
23	1.3	6	1200	40	69.70	7145.65
24	1.5	6	1200	40	67.60	7670.22
25	1.3	6	1200	40	65.40	8012.80
26	1.3	4	1200	40	69.16	6209.50
27	1.1	6	1200	40	64.72	6772.47
28	1.3	8	1200	40	64.68	9153.11
29	1.2	7	1400	60	68.12	2962.03
30	1.3	6	1600	40	67.54	9341.54

**Table 5:** Analysis of variance on micro-hardness.

Source	Sum of Squares		Degree of Freedom	Mean Square		F Value	*p*-Value Prob > F		
Model	1364.86		7	194.98		53.68	<0.0001		Significant
LP	24.77		1	24.77		6.82	0.0159		
SS	4.08		1	4.08		1.12	0.3005		
PR	1243.87		1	1243.87		342.46	<0.0001		
LP × SS	0.42		1	0.42		0.11	0.7382		
LP × PR	6.33		1	6.33		1.74	0.2005		
SS × PR	3.29		1	3.29		0.91	0.3513		
PR^2^	82.11		1	82.11		22.61	<0.0001		
Residual	79.91		22	3.63		-	-		
Lack of Fit	40.37		17	2.37		0.30	0.9719		Not significant
Pure Error	39.54		5	7.91		-	-		
Cor Total	1444.77		29			-	-		
R^2^		0.9447			Adj R^2^			0.9271	
Pred R^2^		0.9068			Adeq Precision			29.479	

**Table 6:** Analysis of variance on wear volume.

Source	Sum of Squares		Degree of Freedom	Mean Square		F Value	*p*-Value Prob > F		
Model	4.321 × 10^8^		7	6.172 × 10^7^		18.81	<0.0001		Significant
LP	1.325 × 10^7^		1	1.325 × 10^7^		4.04	0.0570		
SS	2.790 × 10^6^		1	2.790 × 10^6^		0.85	0.3665		
PR	3.816 × 10^8^		1	3.816 × 10^8^		116.26	<0.0001		
LP×SS	2.148 × 10^7^		1	2.148 × 10^7^		6.54	0.0179		
LP×PR	2.748 × 10^6^		1	2.748 × 10^6^		0.84	0.3701		
SS×PR	8.637 × 10^6^		1	8.637 × 10^6^		2.63	0.1190		
PR^2^	1.583 × 10^6^		1	1.583 × 10^6^		0.48	0.4946		
Residual	7.221 × 10^7^		22	3.282 × 10^6^		-	-		
Lack of Fit	6.774 × 10^7^		17	3.985 × 10^6^		4.46	0.0531		Not significant
Pure Error	4.471 × 10^6^		5	8.942 × 10^5^		-	-		
Cor Total	5.043 × 10^8^		29	-		-	-		
R^2^		0.8568			Adj R^2^			0.8112	
Pred R^2^		0.7008			Adeq Precision			17.048	

**Table 7:** Optimization criteria and limit.

		Criterion	Limit		Importance
			Lower	Upper	
Variable	Laser power	In range	1.1	1.5	3
	Scanning speed	In range	4	8	3
	Gas flow rate	In range	800	1600	3
	TiC powder ratio	In range	0	80	3
Response	Micro-hardness	Target = 62	45.98	75.18	5
	Wear volume	Minimize	518.22	16,519.10	5

**Table 8:** Optimization result and parameter selection of the validation experiment.

	LP (kW)	SS (mm/s)	GF (L/h)	PR (wt.%)	Micro-Hardness	Wear Volume	Desirability	
Prediction	1.5	4	1408.558	26.964	62	3067.37	0.917	Selected
Validation	1.5	4	1400	27	63.16	3261.57	-	-
